# Little White Lies: Pericarp Color Provides Insights into the Origins and Evolution of Southeast Asian Weedy Rice

**DOI:** 10.1534/g3.116.035881

**Published:** 2016-10-10

**Authors:** Yongxia Cui, Beng Kah Song, Lin-Feng Li, Ya-Ling Li, Zhongyun Huang, Ana L. Caicedo, Yulin Jia, Kenneth M. Olsen

**Affiliations:** * College of Science, Sichuan Agricultural University, Ya’an 625014, China; † Department of Biology, Washington University in St Louis, Missouri 63130; ‡ School of Science, Monash University Malaysia, 46150 Bandar Sunway, Selangor, Malaysia; § Key Laboratory of Plant Resources Conservation and Sustainable Utilization, South China Botanical Garden, Chinese Academy of Sciences, Guangzhou 510650, China; ** Department of Biology, University of Massachusetts, Amherst, Massachusetts 01003; †† Dale Bumpers National Rice Research Center, United States Department of Agriculture-Agricultural Research Service, Stuttgart, Arkansas 72160

**Keywords:** adaptive introgression, seed dormancy, targeted genome sequencing, Rc gene, weedy crop relatives

## Abstract

Weedy rice is a conspecific form of cultivated rice (*Oryza sativa* L.) that infests rice fields and results in severe crop losses. Weed strains in different world regions appear to have originated multiple times from different domesticated and/or wild rice progenitors. In the case of Malaysian weedy rice, a multiple-origin model has been proposed based on neutral markers and analyses of domestication genes for hull color and seed shattering. Here, we examined variation in pericarp (bran) color and its molecular basis to address how this trait evolved in Malaysian weeds and its possible role in weed adaptation. Functional alleles of the *Rc* gene confer proanthocyanidin pigmentation of the pericarp, a trait found in most wild and weedy *Oryzas* and associated with seed dormancy; nonfunctional *rc* alleles were strongly favored during rice domestication, and most cultivated varieties have nonpigmented pericarps. Phenotypic characterizations of 52 Malaysian weeds revealed that most strains are characterized by the pigmented pericarp; however, some weeds have white pericarps, suggesting close relationships to cultivated rice. Phylogenetic analyses indicate that the *Rc* haplotypes present in Malaysian weeds likely have at least three distinct origins: wild *O. rufipogon*, white-pericarp cultivated rice, and red-pericarp cultivated rice. These diverse origins contribute to high *Rc* nucleotide diversity in the Malaysian weeds. Comparison of *Rc* allelic distributions with other rice domestication genes suggests that functional *Rc* alleles may confer particular fitness benefits in weedy rice populations, for example, by conferring seed dormancy. This may promote functional *Rc* introgression from local wild *Oryza* populations.

Rice (*Oryza sativa* L.) is one of the world’s most important staple foods, providing the primary calorie source to over one-third of the world’s population (Cheng *et al.* 2003; Khush 1997). One of the major challenges faced by rice farmers worldwide is weedy rice, a conspecific relative of the crop that infests rice fields and aggressively outcompetes desirable cultivars. Weedy rice seedlings are often morphologically nearly identical to cultivated rice plants before they reach the reproductive stage, hindering detection and selective weeding. With a rapid growth rate, highly shattering seed, persistent seed dormancy, and dark, undesirable grains that contaminate harvests, weedy rice is considered one of the primary constraints on rice production and marketability in both temperate and tropical regions (Estorninos *et al.* 2005; Gealy *et al.* 2012; Merotto *et al.* 2016). In addition, the potential for crop–weed gene flow threatens the long-term sustainability of weed control strategies that rely on herbicide-resistant rice cultivars (Pusadee *et al.* 2013; Burgos *et al.* 2014; Merotto *et al.* 2016).

The genetic diversity and origins of weedy rice have been studied extensively, and research to date suggests that weed strains in different world regions have evolved independently from different cultivated rice varieties, including *indica*, *aus*, and *japonica* rice (Reagon *et al.* 2010; Song *et al.* 2014). In Southeast Asia, where rice is grown in close proximity to populations of its wild ancestor, *O. rufipogon*, hybridization with wild populations also appears to have also contributed to the genetic composition of weed populations (Pusadee *et al.* 2013; Song *et al.* 2014). Although weedy rice is specifically adapted to agricultural fields, there are some traits in wild *Oryzas* that would likely be adaptive for the weedy life history strategy if introgressed into nearby weed populations. These include strong seed dormancy, allowing seeds to persist in the soil seed bank over multiple years, as well as increased seed shattering. Both of these traits were selected against during rice domestication and are absent or greatly reduced in domesticated rice varieties. The potential for both wild and domesticated rice to shape the genetic composition of Southeast Asian weed strains makes this region a particularly interesting focus for studying weedy rice evolution (Pusadee *et al.* 2013; Song *et al.* 2014).

In Malaysia, rice fields occupy ∼14% of agricultural lands (Ahmed 2012; Karim *et al.* 2004). Weedy rice was first reported in Malaysia in 1998 (Wahab and Suhaimi 1991), and it has become a major problem over the last two decades (Azmi and Baki 2002), with crop losses in Peninsular Malaysia exceeding $20 million by 2004 (Anuar *et al*. 2014). The proliferation of weedy rice in this region appears to be a direct consequence of shifts to industrialized rice production. Mechanized planting of rice paddies reduces opportunities for detection and hand-weeding in the field; in addition, large-scale commercial rice farming has led to the widespread introduction of elite (high-yielding) modern *indica* cultivars that appear to have given rise to some Malaysian weed strains (Song *et al.* 2014). In an analysis based on 24 nuclear SSRs and sequence variation at two domestication genes (*sh4*, controlling shattering; and *Bh4*, controlling hull color), Song *et al.* (2014) found that both wild and cultivated rice have likely contributed to the composition of contemporary Malaysian weedy rice populations.

Pericarp pigmentation is, together with seed shattering, one of the most defining features of weedy rice (Gross *et al.* 2010a). Most weed strains worldwide possess proanthocyanidin-pigmented (red) pericarps, a phenotype that is characteristic of wild *Oryzas* and associated with persistent seed dormancy (Warwick and Stewart 2005; Gu *et al.* 2011). Pericarp color variation in rice is primarily controlled by the regulatory gene *Rc*, which encodes a bHLH transcription factor that was a genomic target of selection during domestication (Sweeney *et al.* 2007, 2006). The functional (wild-type) *Rc* allele produces a red pericarp; in contrast, most cultivated rice varieties have nonpigmented (white) pericarps, and ∼97% of these carry a loss-of-function *rc* allele characterized by a 14-bp frame shift deletion in exon 7 (Sweeney *et al.* 2007). Besides this predominant *rc* allele, which originated in *japonica* rice and was selectively introgressed into *indica* varieties, an independently evolved domestication allele (*Rc-s*, characterized by a C-to-A nonsense substitution in exon 7) is found specifically in *aus* rice varieties (Sweeney *et al.* 2007, 2006; see also Wang *et al.* 2016). Other *Rc* loss-of-function mutations were selected in the independently domesticated African rice species, *O. glaberrima* (Gross *et al.* 2010b).

The *Rc*-encoded transcription factor pleiotropically regulates both the proanthocyanidin pigment synthesis pathway and abscisic acid-mediated seed dormancy (Gu *et al.* 2011). QTL mapping has indicated that pericarp color variation is correlated with seed dormancy, with *Rc* alleles accounting for 30% of the phenotypic variance in dormancy; germination tests have shown red-pericarp seeds to have 16% lower average germination rates at 7 d (Gu *et al.* 2004, 2011). Thus, the strong selection for *Rc* loss-of-function mutations during rice domestication is likely to reflect both human preferences for nonpigmented grains and selection for crop seeds that readily germinate upon planting (Sweeney *et al.* 2007; Gu *et al.* 2011).

Given its association with seed dormancy, *Rc* is of particular interest in weedy rice evolution (Gross *et al.* 2010a; Li *et al.* 2014; Sweeney *et al.* 2006). For Malaysian weeds, information on the genetic basis of pericarp color variation can provide a useful complement to neutral genetic markers for understanding the relative roles of wild *Oryzas*, recently introduced elite cultivars, and traditional crop landraces in the weed’s evolution. In this study, we examined pericarp color variation and corresponding sequence variation at the *Rc* locus in a set of 52 Malaysian weedy rice accessions. We compared these data to newly generated and previously published *Rc* sequences from wild, cultivated, and United States weedy rice accessions (*N* = 309 *Rc* sequences in total). The aims of our study were to: (1) determine how phenotypic variation in pericarp color in Malaysian weedy rice corresponds to *Rc* sequence variation; (2) assess phylogenetic relationships at *Rc* to draw inferences on evolutionary origins of Malaysian weed haplotypes; and (3) compare patterns observed at *Rc* with three other rice domestication genes, *sh4* (controlling seed shattering), *Bh4* (controlling hull color), and *An*-1 (controlling awn length), to consider the broader adaptive significance of pericarp color variation within Malaysian weedy rice.

## Materials and Methods

### Plant materials and DNA extraction

Data used in this study include newly generated *Rc* sequences and phenotype data, as well as previously published DNA sequences and phenotypes (Gross *et al.* 2010a) (Table 1). The newly generated data are from 156 *Oryza* accessions, including domesticated rice varieties (28 *indica*, 18 *aus*, 12 *japonica*, three *aromatic*), 52 Malaysian weedy rice accessions, 41 *O. rufipogon* accessions (including eight accessions of the annual form, *O. nivara*), and two accessions of the African wild rice species *O. barthii* for use as an outgroup (Supplemental Material, Table S1). The 52 Malaysian weed samples, representing 17 populations distributed across three major rice planting areas of Peninsular Malaysia (northwestern, northeastern, and central-western; Table S1), were collected in 2011 and 2012. Data for pericarp color of wild and cultivated rice were obtained from the online databases of the United States Department of Agriculture (https://npgsweb.ars-grin.gov) and the International Rice Research Institute (IRRI) (http://www.irgcis.irri.org:81/grc/SearchData.htm). Besides the common designations of red or white pericarp, a few cultivated rice accessions are categorized in online databases as has having “brown” or “light brown” pericarps; for those accessions, the alternative designations were included as such. Pericarp color of Malaysian weeds (scored as red or white) was determined by examination of five or more seeds per accession. All 156 rice accessions were grown in the greenhouse at Washington University in St. Louis. Fresh leaf tissue was collected and frozen in liquid nitrogen; DNA was extracted by a modified CTAB procedure (Gross *et al.* 2009).

**Table 1:** Pericarp and *Rc* variations for *O. sativa* and *O. rufipogon* accessions used in this study

Group	Pericarp Color					
	Red (*Rc*)	White		Light Brown (*rc*)	Brown (*Rc-s*)	Mixed (White and Red)
		(*rc*)	(*Rc-s*)			
Domesticated rice (129)						
* indica* (46)	11	29		6		
* aus* (27)	12	4	4		7	
* tropical japonica*						
Asian varieties (24)	10	14				
United States varieties (13)	0	13				
* temperate japonica* (11)	1	8		2		
* aromatic* (8)	0	7	1			
Malaysian weedy rice (52)	43	9				
United States weedy rice (57)						
Black hull awned (24)	24					
Straw hull awnless (24)	24					
Brown hull (5)	5					
Crop–weed hybrids (4)	3	1				
*O. rufipogon* (67)	60	4				3

Sample sizes are indicated in parentheses. Additional details are provided in Table S1 and Table S2.

Previously published data included 153 *Oryza* accessions consisting of domesticated rice (18 *indica*, nine *aus*, 29 *tropical japonica*, seven *temperate japonica*, five *aromatic*); 26 *O. rufipogon* accessions; 57 United States weedy rice strains (comprising 24 straw hull awnless, 24 black hull awned, five brown hull, and four strains of putative crop–weed hybrid origin); and two *O. glumaepatula* outgroup accessions (Table S2).

### DNA sequencing of the Rc genomic region

DNA sequences for the *Rc* genomic region were obtained through targeted genome sequencing using SureSelect (Agilent) technology and Illumina Hi-Seq 2500 sequencing performed at the Whitehead Institute (Massachusetts Institute of Technology). Probes for the *Rc* region were designed based on the rice reference genome (MSU 6.0 assembly). Because the targeted genome sequencing approach failed to reliably detect the *rc* 14-bp deletion (or other indel variations), we designed PCR primers to amplify and direct-sequence the exon 7 region of *Rc* to definitively determine the presence or absence of this functional variation in all Malaysian weedy accessions. Primers were designed using Primer3 (Rozen and Skaletsky 1999), and PCR amplifications were carried out in 20 μl reactions containing the following: 4 μl 5× Promega GoTaq green Flexi Buffer, 2 μl 25 mM MgCl_2_ (2.5 mM in each reaction), 0.4 μl 10 mM dNTPs (200 μM in reaction), 1.0 μl forward and reverse primers (20 μM) respectively, 0.1 μl GoTaq polymerase, and 2 μl genomic DNA template (50 ng/μl). The following PCR profile was used: 2 min at 94° for initial denaturation; followed by 35 cycles of 30 sec at 94°, 30 sec at 55°, and 1 min at 72°; and lastly, 7 min at 72°. DNA sequencing was performed by standard methods on an ABI 3130 capillary sequencer in the Washington University Biology core facility.

### Data analysis

Raw reads from Illumina sequencing were assessed for quality using FastQC software (http://www.bioinformatics.babraham.ac.uk/projects/fastqc/), and the low quality reads were filtered using NGStoolkit (Patel and Jain 2012). Clean reads were mapped onto the reference genome (MSU 6.0 assembly) using BWA (Li and Durbin 2009). Single nucleotide polymorphisms (SNPs) and indels were recorded using SAMtools (Li *et al.* 2009). We then employed a series of Perl scripts to convert the polymorphisms from variant call format (VCF) into FASTA format for sequence alignment. Previously published *Rc* sequences were downloaded from GenBank and combined with our newly obtained data. Sequence alignment was performed using ClustalX (Larkin *et al.* 2007), with editing by hand in Bioedit (Hall 1999) to produce the final alignment.

A maximum likelihood (ML) tree was generated for the *Rc* locus based on high quality SNPs (excluding indels) using the Galaxy website GTR+gamma model (https://usegalaxy.org/root) and the complete dataset consisting of 309 *Oryza* accessions. Indels in the sequence alignment were excluded as they cannot be incorporated in the mutation model employed in this analysis. Statistical support for branches was assessed by bootstrap analysis via 1000 replicated simulations of the dataset. In addition to the ML analysis, we performed a haplotype network analysis with the same SNP dataset using TCS v 1.21 (Clement *et al.* 2000).

Estimates of nucleotide diversity (π and θ*_W_*) were calculated for all accessions and subgroups using DnaSP version 5 (Librado Sanz and Rozas Liras 2009). Tajima's D (Tajima 1989) and Fu and Li's F and D statistics (Fu and Li 1993) were calculated to test for signatures of selection in each group, with significance assessed using 10,000 coalescent simulations and the recombination parameter set to 0. Diversity analyses examined the 6.5 kb *Rc* coding region, as well as a larger 9.7 kb region that included sequences spanning 2.07 kb upstream to 1.17 kb downstream of the *Rc* coding region.

To assess the potential adaptive significance of observed *Rc* allelic distributions within Malaysian weeds, patterns at this gene were compared to functional nucleotide polymorphism (FNP) distributions at three other domestication genes that also control phenotypic variation in cultivated and weedy rice: *sh4* (controlling shattering), *Bh4* (controlling hull color), and *An-1* (controlling awn length). Genotype data for *sh4* and *Bh4* were extracted from Song *et al.* (2014) (*N* = 197 and 185 genotypes for *sh4* and *Bh4*, respectively). For the *An-1* gene, 17 Malaysian weed accessions were selected to represent plants that vary in awn length. The following *An-1* PCR primers were designed to amplify and sequence a 775-bp FNP region previously identified by Luo *et al.* (2013), spanning exons 1 and 2: forward primer An-01F, 5′-AGCGCCAACAACTCCTGCTAC-3′; reverse primer An-01R, 5′-GCTTCATCCTCTCGCTTATCCTC-3′. Amplified products were sequenced directly using Sanger sequencing (ABI PRISM BigDye Terminator Cycle Sequencing Reaction Kit, Perkin Elmer) at the First BASE Laboratories Sdn. Bhd. (Malaysia). Accessions were scored for three FNPs identified by Luo *et al.* (2013): a GCC/– – – deletion, a C/G substitution, and a G/– deletion.

### Data availability

Newly generated DNA sequences are available in GenBank (accession nos. KX549104–KX549259). Table S1 contains IDs, phenotypes, and genotypes of newly characterized accessions, including *Rc* genotypes confirmed by direct sequencing of exon 7 and *An-1* genotype data. Table S2 contains previously published data included in analyses. The *Rc* alignment used in phylogeny construction is available on Dryad (doi: 10.5061/dryad.631kf).

## Results

### Distribution of Rc alleles and pericarp color variation

Malaysian weedy rice accessions were variable with respect to pericarp color, with 43 red-pericarp and 9 white-pericarp accessions in the sample set (Table 1 and Table S1). This stands in contrast to United States weeds, where the red pericarp is nearly universally present (Gross *et al.* 2010a) and the weed is commonly referred to as “red rice.” Most domesticated rice varieties in the sample set possessed white pericarps (although sample selection was intentionally enriched to represent red-pericarp varieties), and most wild rice (*O. rufipogon*) had red pericarps; this pattern was consistent with previous studies (Sweeney *et al.* 2006, 2007; Gross *et al.* 2010a) (Table S2).

The length of the aligned *Rc* dataset generated by targeted genome sequencing is 9773 bp. This spans 2068 bp upstream of the start codon, the entire 6528 bp coding region, and 1177 bp downstream of the stop codon. Consistent with previous findings, all white-pericarp (and light brown pericarp) accessions of cultivated rice were found to carry one of the two previously reported *O. sativa* loss-of-function alleles (*rc* and *Rc-s*), of which the rare *Rc-s* allele was only observed in four *aus* and one *aromatic* accession (Table 1). In addition, as previously reported, all United States weedy rice strains carried putatively functional *Rc* alleles (Gross *et al.* 2010a). Malaysian weeds carried both functional *Rc* sequences and the *rc* loss-of-function allele, consistent with the phenotypic variation observed in this group.

**Figure 1:**
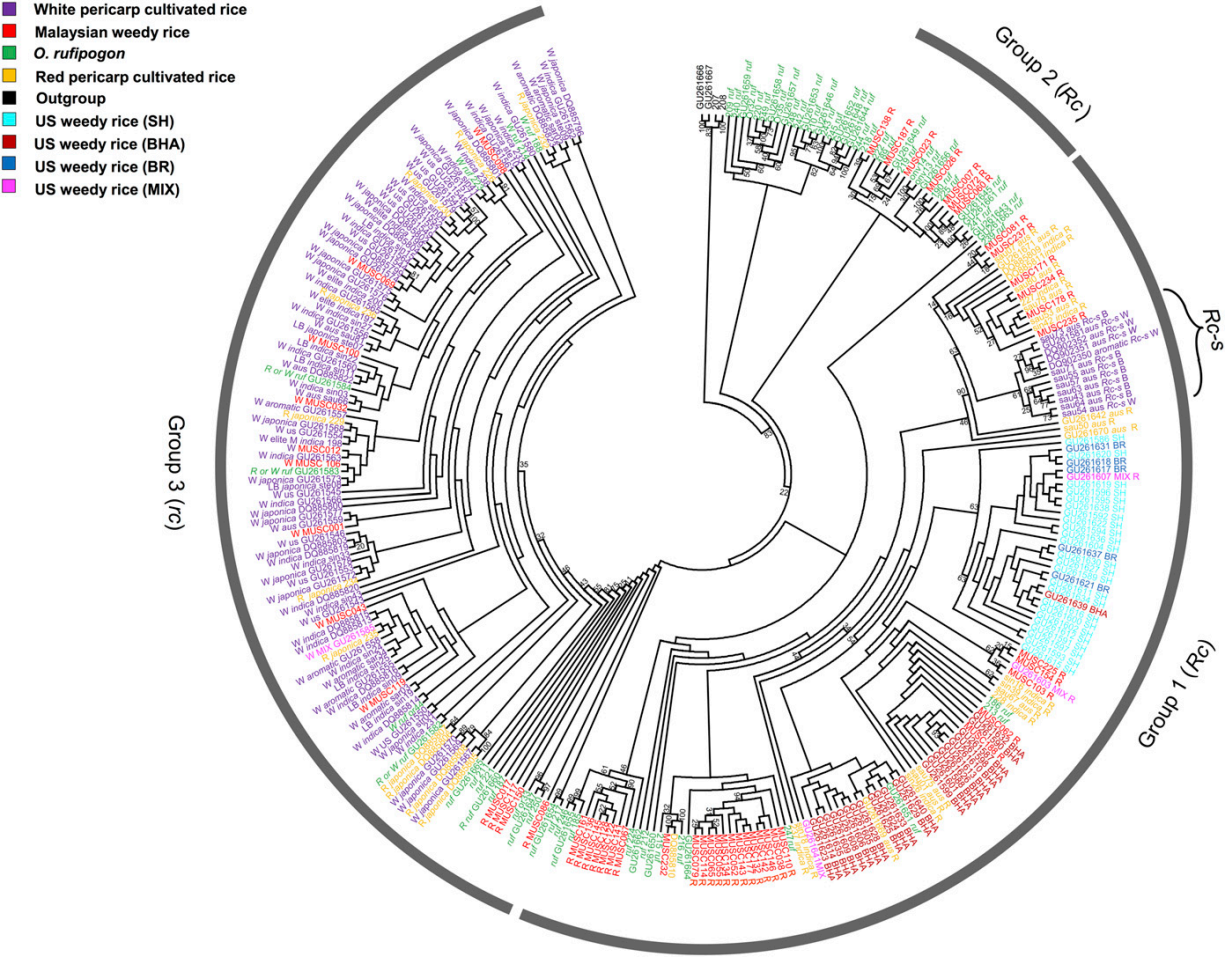
Maximum likelihood (ML) tree of *Rc* haplotypes. Different colors indicate identity of accessions, as indicated in the key. Numbers on branches indicate bootstrap support. BHA, black hull awned; BR, brown hull; MIX, crop–weed hybrid; SH, straw hull awnless.

To examine the phylogenetic origin of pericarp color variation in Malaysian weedy rice, we performed ML tree construction and TCS haplotype network analyses using a combined dataset of the newly generated and previously published *Rc* sequences; the aligned dataset contained 721 SNPs with indels excluded (Dryad doi: 10.5061/dryad.631kf). On the resulting ML tree, Malaysian weedy rice accessions fall into three phylogenetically distinct groups (Figure 1); this pattern is also evident in the TCS haplotype network (Figure S1). The largest group of Malaysian weeds (32 of 52 accessions) occur in a large clade (labeled group 1) where they are clustered with United States weeds, red-pericarp domesticated rice (12 *aus* and 11 *indica* varieties), white- or brown-pericarp domesticated rice varieties that carry the *Rc-s* nonsense mutation (11 *aus* and one *aromatic* variety), and a few *O. rufipogon* accessions. A second clade (group 2) contains seven Malaysian weeds that are grouped exclusively with *O. rufipogon* accessions. The third group of Malaysian weeds (group 3) is characterized by haplotypes that either carry the *rc* 14-bp deletion or have functional *Rc* sequences closely related to *rc* haplotypes. All white-pericarp Malaysian weeds occur in this group.

Direct Sanger sequencing of the *Rc* exon 7 region confirmed the presence of the *rc* 14-bp frame shift deletion in eight of the nine white-pericarp Malaysian weeds. The one exception (MUSC032) showed no other obvious evidence of an independent loss-of-function mutation in the *Rc* gene. This genotype–phenotype discrepancy may reflect an instance of heterozygosity at the *Rc* locus in this weed strain, such that the individual plant grown for DNA sequencing (potentially a red-pericarp *Rc/rc* heterozygote) differed genetically from the seed material used in assessing pericarp color (potentially a white-pericarp *rc* homozygote). Recent hybridization between plants carrying *Rc* and *rc* alleles could account for such heterogeneity. Consistent with this hypothesis, all four red-pericarp Malaysian weeds that are clustered in group 3 were revealed by Sanger sequencing to be heterozygotes for the *rc* mutation (Table S1), which further suggests recent hybridization between plants with functional and nonfunctional *Rc* alleles.

Taken together, the haplotype groupings in the ML tree and TCS network suggest that the *Rc* sequences present in Malaysian weed strains are derived from at least three distinct sources: wild *O. rufipogon* populations (most evident for accessions in group 2); white-pericarp domesticated rice (group 3); and red-pericarp *indica* and *aus* rice varieties that did not undergo selection for the white-pericarp phenotype found in most contemporary rice (group 1). Thus, it appears that reproductively compatible *O. rufipogon* populations, together with phenotypically diverse domesticated rice varieties, have contributed to the genetic and phenotypic complexity of Malaysian weeds.

In order to determine which SNPs contribute to the *Rc* haplotype differentiation between the three groups, we examined the nucleotide variation in the genomic region spanning 2068 bp upstream of the start codon to 1177 bp downstream of the stop codon in the Malaysian weed accessions. For white-pericarp Malaysian weeds (group 3), there are 13 SNPs that are diagnostic of this group, in comparison to two unique SNPs for group 1 (containing most weed accessions) and no unique SNPs for the *O. rufipogon*-like group 2 (Table S3). The greater differentiation of group 3 haplotypes from the other two groups is consistent with the known origin of the *rc* allele from a *japonica* background (Sweeney *et al.* 2007), which would be expected to show high differentiation from the *indica*- and *aus*-related accessions occurring in groups 1 and 2 (Figure 1). Multiple sequence alignment of the *Rc* coding region and predicted amino acid sequences confirmed that the *rc* 14-bp deletion occurs specifically in white-pericarp cultivated rice and Malaysian weedy rice (genomic sequence positions 1429–1442 bp; Table S4). Among the 24 SNPs within exon regions, two are nonsynonymous, one of which is predicted to produce a Q-to-H gain-of-charge amino acid replacement (position 21; Table S4). This replacement occurs in Malaysian weeds and *indica* varieties in group 3, as well as one Malaysian weed in group 1. Both of the nonsynonymous substitutions occur outside the functionally critical DNA-binding HLH domain (Sweeney *et al.* 2006), however, and may have no phenotypic effect.

### Genetic diversity analysis

Artificial selection during domestication is expected to decrease the nucleotide diversity at the genomic target of selection, as the favored domestication allele rises to high frequency and displaces neutral variation. To examine changes in nucleotide variation at the *Rc* locus, we calculated the nucleotide diversity of wild, cultivated, and weedy rice. As expected, white-pericarp cultivated rice (carrying the *rc* allele) shows reduced nucleotide diversity (silent-site π = 0.04/kb) compared to *O. sativa* overall (silent-site π = 0.95/kb) and the wild progenitor *O. rufipogon* (silent-site π = 1.30/kb) (Table 2; see also Table S5). Correspondingly, the Tajima’s *D* value of white-pericarp cultivated rice shows a statistically significant deviation from neutrality in a direction suggesting strong positive selection for the domestication allele (*D* = −2.283; *P* < 0.01). Red-pericarp crop varieties, which were not subject to such selection, show no significant deviation from neutrality at *Rc*. These results are consistent with previous findings of selection signatures at *Rc* for the white-pericarp domestication phenotype (Gross *et al.* 2010a; Sweeney *et al.* 2007, 2006). Interestingly, the wild progenitor, *O. rufipogon*, also shows a significantly negative Tajima’s *D* value (*D* = −2.570; *P* < 0.001), despite most of the wild accessions possessing red pericarps and functional *Rc* alleles; in this case, however, the deviation reflects the low-frequency presence of *rc* alleles in wild rice, which generate a statistical excess of rare SNPs in the sample set (Table S4).

**Table 2:** Nucleotide diversity and Tajima’s *D* at the *Rc* locus in wild, cultivated, and Malaysian weedy rice

	*O. rufipogon*	*O. sativa*			Malaysian Weedy Rice				
	(*n* = 67)	All Cultivated Rice (*n* = 129)	White Pericarp Cultivated Rice (*rc*)*^a^* (*n* = 83)	Red Pericarp Cultivated Rice (*n* = 34)	All Malaysian Weeds (*n* = 52)	White Pericarp Malaysian Weeds (*n* = 9)	Malaysian Weeds in Group 1 (*n* = 32)	Malaysian Weeds in Group 2 (*n* = 7)	Malaysian Weeds in Group 3 (*n* = 13)
π per kb (silent sites)	1.30	0.95	0.04	2.57	1.44	0.31	1.50	4.01	0.12
π per kb (all sites)	1.13	0.87	0.04	2.26	1.24	0.26	1.28	3.47	0.10
θ_w_ per kb (silent sites)	5.06	1.44	0.30	2.21	2.66	0.51	1.51	4.30	0.08
θ_w_ per kb (all sites)	4.34	1.33	0.30	1.81	2.35	0.44	1.34	3.72	0.07
Tajima’s *D*	−2.5700***	−1.0633	–2.2830**	−0.91202	−1.6765	–1.8613*	−0.1526	0.3886	−0.1526

***, **, and * for Tajima’s *D* values indicates statistical significance at *P* < 0.001, *P* < 0.01, and *P* < 0.05, respectively.

a Includes varieties scored as “light brown” in online databases.

Like domesticated rice, white-pericarp Malaysian weeds also show reductions in *Rc* nucleotide diversity and a statistically significant deviation from neutrality in a direction consistent with selection for the *rc* allele (silent-site π = 0.31/kb; Tajima’s *D* = −1.861; *P* < 0.05) (Table 2 and Table S5). However, most of the weed strains do not have this domestication phenotype, and nucleotide diversity for Malaysian weeds overall (silent site π = 1.44/kb) exceeds that of all cultivated *O. sativa*, and is even marginally higher than the wild species *O. rufipogon* (silent site π = 1.30/kb). The high *Rc* nucleotide diversity present in these weeds supports our conclusion based on phylogenetic analyses that Malaysian weed haplotypes have diverse origins from both cultivated and wild rice.

**Figure 2:**
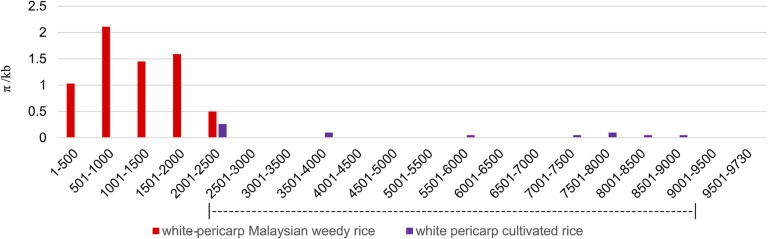
Comparison of nucleotide diversity across the *Rc* genomic region for white-pericarp Malaysian weedy rice *vs.* white-pericarp cultivated rice. Diversity is shown for accessions that carry the *rc* allele, using 500-bp windows across the 5′ upstream region (0–2 kb), the *Rc* coding region (2–8.5 kb), and the 3′ downstream region (8.5–9.7 kb). The last window is 230 bp. The dashed line corresponds to the approximate location of the *Rc* coding region.

To further explore the distribution of genetic diversity at the *Rc* locus, we compared nucleotide diversity across the larger 9.7 kb region (spanning upstream and downstream sequences) for white-pericarp weeds with cultivated rice accessions carrying the *rc* allele. Despite the shared presence of the *rc* allele within the coding region, the white-pericarp weeds show higher genetic diversity in the immediate 5′ flanking sequence (Figure 2). This elevated diversity is attributable to a single weed accession, MUSC098, which carries an upstream sequence that differs from the other white-pericarp weed accessions by 15 SNPs, all of which are shared with red-pericarp weed accessions (Table S3). This pattern suggests that MUSC098 carries a recombinant haplotype, with the *rc* allele in the coding region and a sequence characteristic of red-pericarp weeds in its upstream sequence.

### Disproportionate representation of wild Rc alleles in Malaysian weeds

**Figure 3:**
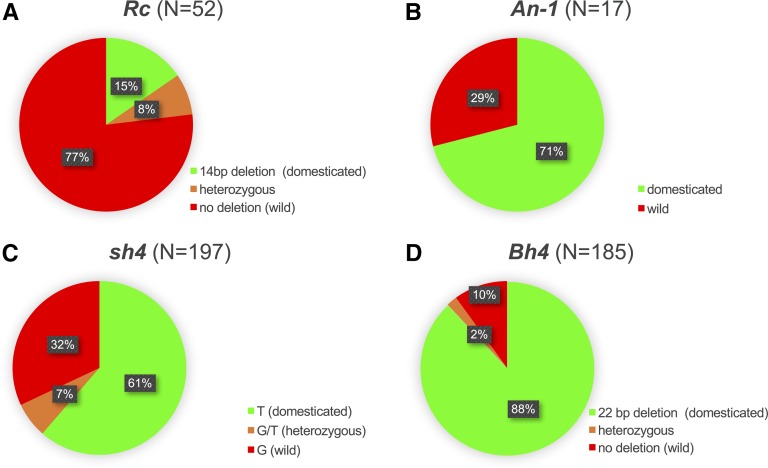
Percentage of Malaysian weedy rice accessions with wild-type *vs.* domestication alleles for four domestication genes: (A) *Rc*, (B) *An-1*, (C) *sh4*, and (D) *Bh4*. *N* indicates numbers of accessions genotyped per gene. Genotyping details for each sampled accession are listed in Table S1. Data for *sh4* and *Bh4* were extracted from Song *et al.* (2014).

*Rc* differs markedly from the other domestication genes examined in the proportions of wild *vs.* domestication alleles represented in Malaysian weeds. For *An-1* (controlling awn length), *Bh4* (controlling hull color), and *sh4* (controlling seed shattering), the majority of Malaysian weeds are homozygous for domestication alleles (ranging from 61% of accessions for *sh4* to 88% for *Bh4*; Figure 3 and Table S1). This preponderance of domestication alleles is consistent with previous inferences that domesticated rice has played a major role in the evolution of Malaysian weeds (Song *et al.* 2014). In contrast, for *Rc* the opposite pattern is observed: the majority of Malaysian weeds (77%) are homozygous for the wild-type functional allele that is characteristic of wild *Oryzas* and absent in the vast majority of modern cultivated rice varieties (Figure 3A). This *Rc*-specific pattern suggests that selection may be playing a role in elevating *Rc* allele frequencies in Malaysian weedy rice. Given the known functional importance of *Rc* in maintaining rice seed dormancy (Gu *et al.* 2011; Subudhi *et al.* 2012), this disproportionate occurrence of wild-type *Rc* alleles could potentially reflect selection to maintain seed dormancy alleles introgressed from wild *O. rufipogon* into Malaysian weed populations.

## Discussion

As one of the primary genetic determinants of pericarp color, *Rc* was a target of strong selection during rice domestication because humans selected for nonpigmented grains (Sweeney *et al.* 2007, 2006). Given the pleiotropic role of the *Rc*-encoded transcription factor in regulating seed dormancy (Gu *et al.* 2011), this selective event may also reflect human selection for crop seeds that readily germinate upon sowing. In contrast to cultivated rice, seed dormancy is highly adaptive in agricultural weeds, as it allows seeds to persist in crop fields over multiple seasons (Delouche and Labrada 2007). Here, we find evidence that in Malaysian weedy rice, *Rc* allelic diversity has likely been enhanced by both wild *O. rufipogon* and cultivated rice (Figure 1, Table 2, and Figure S1), and that selection may specifically favor elevated frequencies of functional wild-type *Rc* alleles in weed populations (Figure 3), which would be expected to confer seed dormancy. Below we discuss these findings and their potential implications for weedy rice evolution and adaptation.

### Contributors to Rc diversity in Malaysian weedy rice

#### Oryza rufipogon:

The *Rc* locus has been studied extensively in cultivated rice (Gulick *et al.* 2009; Sweeney *et al.* 2007, 2006; Wang *et al.* 2016) and to a lesser extent in United States weedy rice (Gross *et al.* 2010a); it has not, however, previously been examined in weedy rice populations that grow in regions where they can be influenced by gene flow from local wild *Oryzas*. For Malaysian weeds, which often occur in proximity to wild *O. rufipogon*, the likely influence of wild populations is evident in the *Rc* phylogenetic analysis, where we detect *Rc* haplotypes that are characteristic of the wild species in the weeds (*e.g.*, group 2, Figure 1; see also Figure S1), and in our comparison across domestication genes, where we find disproportionate representation of wild-type alleles compared to other domestication genes (Figure 3). Notably, more than three-quarters (77%) of weed accessions in our sample set carry functional *Rc* alleles, a marked contrast from allelic distributions at the domestication genes *sh4*, *Bh4*, and *An-1*. In a recent morphology based study of a larger sampling of Malaysian weedy rice (*N* = 193 accessions), Sudinato *et al.* (2016) observed red or brown pericarp color in a comparable proportion of the samples (68%). This suggests that *Rc* allelic distribution observed here may be generally representative of Malaysian weed populations.

#### Red-pericarp rice:

Unlike domestication genes such as the shattering locus *sh4*, where cultivated rice worldwide is nearly 100% fixed for the domestication allele, domestication alleles of *Rc* are not completely fixed in cultivated rice, and while rare nowadays, some red-pericarp Malaysian landraces are still grown that carry functional *Rc* haplotypes. A proportion of the functional *Rc* alleles observed in Malaysian weeds may therefore be derived from red-pericarp crop landraces rather than *O. rufipogon*. Weed haplotypes in *Rc* group 1 are the most likely candidates to be of crop origin, as most red-pericarp rice varieties cluster within this group (see Figure 1 and Figure S1). Cultivation of red-pericarp landraces in Malaysia has declined to very low levels in recent decades, particularly following the industrialization of rice production. No red-pericarp landraces have been grown in any of the locations sampled for the present study for at least 30 yr (B. K. Song, unpublished data), and *aus* rice varieties (which make up the majority of red-pericarp rice landraces) do not occur in Malaysia. This suggests that red-pericarp landraces probably play a limited role, if any, in the contemporary evolution of Malaysian weed populations, although they may have played a more important role in the past (see Song *et al.* 2014).

#### White-pericarp rice:

Whereas red-pericarp cultivated rice may have a limited role in ongoing Malaysian weed evolution, the white-pericarp varieties are clearly influencing contemporary phenotypic and genetic diversity of the weed. Nine of the 52 sampled accessions (17%) have white pericarps, eight of which were confirmed to carry the 14-bp deletion *rc* allele (Figure 1 and Table 1). Moreover, direct Sanger sequencing revealed that four additional red-pericarp weeds carry the *rc* allele in a heterozygous state (Table S1). Like cultivated rice, weedy rice is predominantly self-fertilizing, and heterozygosity would be expected to decline to negligible levels within just a few generations of a hybridization event. The observation here of four *Rc*/*rc* heterozygotes, along with the substantial proportion of white-pericarp weeds (Table S1), strongly suggests that crop–weed hybridization is contributing to the genetic diversity of Malaysian weedy rice, and that this is an ongoing process.

While *Rc* sequences do not provide insight on which specific crop varieties are most likely to be contributing *rc* alleles to Malaysian weedy rice, a complementary analysis based on neutral SSR markers has implicated recently introduced elite cultivars in the weed’s evolution (Song *et al.* 2014). The study revealed some Malaysian weed strains to be genetically highly similar to elite *indica* varieties; these modern cultivars were originally developed at the IRRI (Dalrymple 1986) and were later mass-distributed in Malaysia with the advent of industrialized rice production.

It should be noted that because the *Rc* alignment excluded indels (which could be scored less reliably from genome sequence data; see *Materials and Methods*), the *rc* 14-bp deletion was not included as a phylogenetic character in generating the ML tree, and the presence or absence of this mutation was treated as unknown for previously published *Rc* sequences (Table S1). The *rc* allele is known to have a single mutational origin from a *japonica* rice background (Sweeney *et al.* 2007), and as such, the haplotypes that carry this mutation (characterizing group 3 sequences; Figure 1 and Figure S1) differ by multiple SNPs from the *indica*, *aus*, and *O. rufipogon* sequences that characterize groups 1 and 2. All of the accessions in groups 1 and 2 for which we have phenotypic data are characterized by red pericarps (consistent with carrying functional *Rc* alleles), or they have white pericarps resulting from the previously described *Rc-s* nonsense mutation (Table S1). Thus, it appears unlikely that any of the previously published accessions within groups 1 and 2 would carry the 14-bp *rc* deletion.

#### Consequences for genetic and phenotypic diversity:

The cumulative effects of these multiple contributors to Malaysian weedy rice are evident not only in the *Rc* phylogeny (Figure 1 and Figure S1) but also in levels of nucleotide diversity at this locus. For both silent sites and all sites across the *Rc* gene, the average pairwise nucleotide diversity (π) of the Malaysian weeds exceeds that of the cultivated rice samples, and even exceeds that of a geographically wide sample of wild rice (*O. rufipogon*) (Table 2 and Table S5). This high diversity provides a particularly striking contrast to *Rc* variation previously observed in United States weedy rice (Gross *et al.* 2010a), where the reported nucleotide diversity (π/kb = 0.34 and 0.41 for total and silent sites, respectively) is <30% of the values observed here. This large difference in *Rc* diversity between United States and Malaysian weeds is likely to reflect not only the more dynamic crop–weed gene flow interactions that can occur in Southeast Asia, but also the sharp demographic bottleneck that occurred with the introduction of weedy rice into North America from Asia (Reagon *et al.* 2010).

### Rc and weedy rice adaptation

The disproportionate representation of functional (wild-type) *Rc* alleles in Malaysian weedy rice compared to other domestication genes (Figure 3) is consistent with *Rc* serving an adaptive function in weed populations. Pericarp pigmentation could conceivably provide camouflage for weed seeds in crop fields (Vigueira *et al.* 2013 a,b), or protection from abiotic and biotic stress (Ithal and Reddy 2004; Martínez-Castillo *et al.* 2008). However, the adaptive value of functional *Rc* alleles may be more likely to come from the gene’s effects in maintaining seed dormancy. The ability of agricultural weed seeds to remain dormant in the soil over multiple planting seasons plays a key role in their persistence and proliferation (Delouche and Labrada 2007; Vigueira *et al.* 2013b), and the *Rc* locus has been identified as one of the two major effect QTL for seed dormancy in United States weedy rice (Subudhi *et al.* 2012). While the functional effects of *Rc* sequence variation on seed dormancy have not been explicitly examined, it is reasonable to expect that wild-type sequences that can successfully regulate proanthocyanidin synthesis would also be functional in establishing seed dormancy. Phenotypic assays of seed dormancy in the Malaysian weeds would be useful for testing this prediction.

If functional *Rc* alleles are, as proposed, adaptive for seed dormancy in Malaysian weed populations, the question then arises as to why 17% of the sampled accessions were homozygous for the loss-of-function *rc* allele. One possibility is that these accessions represent recently derived, maladapted descendants of crop–weed hybridization that are unlikely to persist over multiple generations. The observation of four *Rc/rc* heterozygotes in these highly selfing weeds supports the hypothesis that the crop-derived *rc* allele is of recent origin in the weeds, and may be only transiently present. Studies that explicitly examine the relationships between *Rc* sequence variation, gene expression, phenotypic variation (both seed dormancy and pericarp color), and fitness in field plantings would be valuable for definitively establishing the adaptive role of *Rc* alleles in weedy rice.

### Malaysian weedy rice: a Trojan horse for contamination of crop and wild germplasm?

Our inferences for the *Rc* locus, together with previous neutral-marker analyses (Song *et al.* 2014), strongly suggest that both cultivated and wild rice populations are contributing to the genetic composition of Malaysian weedy rice through hybridization, and that this may be contributing to its adaptation and rapid proliferation. If Malaysian weeds are, in fact, a crucible where crop and wild alleles can recombine, the weeds could also potentially serve as a bridge for the introduction of crop alleles into wild populations (Snow *et al.* 2010; Campbell *et al.* 2016) or vice versa (Félix *et al.* 2014). Of these two gene flow possibilities, wild-to-crop gene flow may be the less serious problem. Although in principle, the proximity of weedy rice to crops within rice fields could allow them to serve as a Trojan horse for the introgression of undesirable wild traits, crop seed-stock certification can be expected to minimize the contamination of rice germplasm. More problematic is the possibility of genetic swamping of the wild progenitor populations (*e.g.*, Martínez-Castillo *et al.* 2008; Cornille *et al.* 2015; Fuchs *et al.* 2016), which, in the case of Malaysian *O. rufipogon*, are increasingly rare and have no *in situ* conservation status. Introgression from cultivated rice into weedy rice is not uncommon (Xia *et al.* 2011; Ellstrand *et al.* 2013; Jiang *et al.* 2012), and the recent widespread adoption of herbicide-resistant rice production will likely increase hybridization due to selection for introgression of resistance alleles into weed populations (Burgos *et al.* 2014). Combined with the recent demographic explosion in weedy rice populations across Southeast Asia (Azmi *et al.* 2013; Pusadee *et al.* 2013), weedy rice-mediated genetic erosion of wild *O. rufipogon* may become an increasing concern in this region.

### Conclusions

Our results show that weedy rice in Peninsular Malaysia appears to have diverse origins, resulting in high *Rc* genetic diversity in the weeds. The multiple-origin model of *Rc* alleles in Malaysian weedy rice is in line with co-occurrence of cultivated and wild rice at the edges of rice fields in this region. This is also consistent with the previous analyses of domestication genes of Malaysian weedy populations, where multiple origins were demonstrated for alleles of the *sh4* and *Bh4* domestication genes (Song *et al.* 2014). The disproportionate representation of functional *Rc* alleles in Malaysian weeds compared to other domestication genes suggests that crop–weed gene flow may differentially promote high frequencies of functional *Rc* alleles. With the potential disadvantageous features incurred by the domestication *rc* allele (*e.g.*, lack of seed dormancy), we predict that the nonfunctional *rc* alleles may be purged from the weedy populations unless continued crop–weed hybridization maintains their presence. Future work is needed, however, to assess the relationship between *Rc* expression and its full range of phenotypic and fitness effects. A follow-up study should also consider whether and how crop–weed introgression may differentially shape allele frequencies at other rice domestication genes. The evidence that red-pericarp cultivated rice, possibly the traditional landraces planted before the cultivation of modern elite varieties, may be one of the potential origins of weedy rice should be an alarming signal to the rice industry. A practical implication of our work is that more stringent seed-stock certification guidelines should be in place in local rice breeding programs, in order to avoid severe and long-term impacts that could compromise crop productivity.

## Supplementary Material

Supplemental Material

## References

[bib1] Ahmed Q. N., 2012 Vegetative and reproductive growth of weedy rice in Selangor, Malaysia: a comparative study with commercial rice varieties. Malaysian Applied Biology 41: 29–35.

[bib50] Anuar N. H. S. Mazlan N. Ariff E. A. K. E. Juraimi A. S. Yusop M. R., 2014 A comparative study of vegetative and reproductive growth of local weedy and Clearfield® rice varieties in Malaysia. J. ISSAAS. 20: 41–51.

[bib2] Azmi, M., and B. Baki, 2002 Impact of continuous direct seeding rice culture on weed species diversity in the Malaysian rice ecosystem. *Proceedings of Regional Symposium on Environment and Natural Resources* (Kuala Lumpur, Malaysia April 10–11, 2002), Universiti Kebangsaan Malaysia, Bangi, Vol. 1, pp. 61–67.

[bib3] Burgos N. R. Singh V. Tseng T. M. Black H. Young N. D., 2014 The impact of herbicide-resistant rice technology on phenotypic diversity and population structure of United States weedy rice. Plant Physiol. 166: 1208–1220. 25122473 10.1104/pp.114.242719 PMC4226343

[bib4] Campbell L. G. Lee D. Shukla K. Waite T. A. Bartsch D., 2016 An ecological approach to measuring the evolutionary consequences of gene flow from crops to wild or weedy relatives. Appl. Plant Sci. 4: 1500114. 10.3732/apps.1500114 PMC4795919 27011898

[bib5] Cheng C. Motohashi R. Tsuchimoto S. Fukuta Y. Ohtsubo H., 2003 Polyphyletic origin of cultivated rice: based on the interspersion pattern of SINEs. Mol. Biol. Evol. 20: 67–75. 12519908 10.1093/molbev/msg004

[bib6] Clement M. Posada D. Crandall K. A., 2000 TCS: a computer program to estimate gene genealogies. Mol. Ecol. 9: 1657–1659. 11050560 10.1046/j.1365-294x.2000.01020.x

[bib7] Cornille A. Feurtey A. Gélin U. Ropars J. Misvanderbrugge K., 2015 Anthropogenic and natural drivers of gene flow in a temperate wild fruit tree: a basis for conservation and breeding programs in apples. Evol. Appl. 8: 373–384. 25926882 10.1111/eva.12250 PMC4408148

[bib8] Dalrymple D. G., 1986 *Development and Spread of High-Yielding Rice Varieties in Developing Countries*. Agency for International Development, Washington, DC.

[bib9] Delouche J. C. Labrada R., 2007 Weedy rices: origin, biology, ecology and control. Food Agr. Org. 188: 45–62.

[bib10] Ellstrand N. C. Meirmans P. Rong J. Bartsch D. Ghosh A., 2013 Introgression of crop alleles into wild or weedy populations. Annu. Rev. Ecol. Evol. Syst. 44: 325–345.

[bib11] Estorninos L. E. Gealy D. R. Talbert R. E. Gbur E. E., 2005 Rice and red rice interference. I. Response of red rice (*Oryza sativa*) to sowing rates of tropical *japonica* and *indica* rice cultivars. Weed Sci. 53: 676–682.

[bib12] Félix D. T. Coello-Coello J. Martínez-Castillo J., 2014 Wild to crop introgression and genetic diversity in lima bean (*Phaseolus lunatus L*.) in traditional Mayan milpas from Mexico. Conserv. Genet. 15: 1315–1328.

[bib51] Fu Y.-X. Li W.-H. 1993 Statistical tests of neutrality of mutations. Genetics. 133: 693–709. 8454210 10.1093/genetics/133.3.693 PMC1205353

[bib13] Fuchs E. J. Martínez A. M. Calvo A. Muñoz M. Arrieta Espinoza G., 2016 Genetic diversity in *Oryza glumaepatula* wild rice populations in Costa Rica and possible gene flow from *O. sativa*. PeerJ 4: e1875. 27077002 10.7717/peerj.1875 PMC4830232

[bib14] Gealy D. R. Agrama H. Jia M. H., 2012 Genetic analysis of atypical US red rice phenotypes: indications of prior gene flow in rice fields? Weed Sci. 60: 451–461.

[bib15] Gross B. L. Skare K. J. Olsen K. M., 2009 Novel *Phr1* mutations and the evolution of phenol reaction variation in US weedy rice (*Oryza sativa* L.). New Phytol. 184: 842–850. 19674331 10.1111/j.1469-8137.2009.02957.x PMC2847516

[bib16] Gross B. L. Reagon M. Hsu S. C. Caicedo A. L. Jia Y., 2010 a Seeing red: the origin of grain pigmentation in US weedy rice. Mol. Ecol. 19: 3380–3393. 20584133 10.1111/j.1365-294X.2010.04707.x PMC2921015

[bib17] Gross B. L. Steffen F. T. Olsen K. M., 2010 b Molecular basis of white pericarps in African domesticated rice: novel mutations at the *Rc* gene. J. Evol. Biol. 23: 2747–2753. 21121088 10.1111/j.1420-9101.2010.02125.x PMC3058869

[bib18] Gu X. Y. Kianian S. F. Foley M. E., 2004 Multiple loci and epistases control genetic variation for seed dormancy in weedy rice (*Oryza sativa*). Genetics 166: 1503–1516. 15082564 10.1534/genetics.166.3.1503 PMC1470771

[bib19] Gu X. Y. Foley M. E. Horvath D. P. Anderson J. V. Feng J., 2011 Association between seed dormancy and pericarp color is controlled by a pleiotropic gene that regulates abscisic acid and flavonoid synthesis in weedy red rice. Genetics 189: 1515–1524. 21954164 10.1534/genetics.111.131169 PMC3241415

[bib20] Gulick P. Lee D. Lupotto E. Powell W., 2009 G-string slippage turns white rice red. Genome 52: 490–493. 19448729 10.1139/g09-025

[bib21] Hall T. A., 1999 BioEdit: a user-friendly biological sequence alignment editor and analysis program for Windows 95/98/NT. Nucleic Acids Symp. Ser. 41: 95–98.

[bib22] Ithal N. Reddy A. R., 2004 Rice flavonoid pathway genes, *OsDfr* and *OsAns*, are induced by dehydration, high salt and ABA, and contain stress responsive promoter elements that interact with the transcription activator, OsC1-MYB. Plant Sci. 166: 1505–1513.

[bib23] Jiang Z. Xia H. Basso B. Lu B. R., 2012 Introgression from cultivated rice influences genetic differentiation of weedy rice populations at a local spatial scale. Theor. Appl. Genet. 124: 309–322. 21947325 10.1007/s00122-011-1706-5

[bib24] Karim R. S. Man A. B. Sahid I. B., 2004 Weed problems and their management in rice fields of Malaysia: an overview. Weed Biol. Manage. 4: 177–186.

[bib25] Khush G. S., 1997 Origin, dispersal, cultivation and variation of rice. Plant Mol. Biol. 35: 25–34. 9291957

[bib26] Larkin M. A. Blackshields G. Brown N. P. Chenna R. McGettigan P. A., 2007 Clustal W and Clustal X version 2.0. Bioinformatics 23: 2947–2948. 17846036 10.1093/bioinformatics/btm404

[bib27] Li H. Durbin R., 2009 Fast and accurate short read alignment with Burrows-Wheeler transform. Bioinformatics 25: 1754–1760. 19451168 10.1093/bioinformatics/btp324 PMC2705234

[bib28] Li H. Handsaker B. Wysoker A. Fennell T. Ruan J., 2009 The sequence alignment/map format and SAMtools. Bioinformatics 25: 2078–2079. 19505943 10.1093/bioinformatics/btp352 PMC2723002

[bib29] Li X. Qiang S. Song X. Cai K. Sun Y., 2014 Allele types of *Rc* gene of weedy rice from Jiangsu Province, China. Rice Sci. 21: 252–261.

[bib30] Librado Sanz P. Rozas Liras J. A., 2009 DnaSP v5: a software for comprehensive analysis of DNA polymorphism data. Bioinformatics 25(11): 1451–1452. 19346325 10.1093/bioinformatics/btp187

[bib31] Luo J. Liu H. Zhou T. Gu B. Huang X., 2013 *An-1* encodes a basic helix-loop-helix protein that regulates awn development, grain size, and grain number in rice. Plant Cell 25: 3360–3376. 24076974 10.1105/tpc.113.113589 PMC3809537

[bib32] Martínez-Castillo J. Colunga-GarcíaMarín P. Zizumbo-Villarreal D., 2008 Genetic erosion and *in situ* conservation of lima bean (*Phaseolus lunatus* L.) landraces in its Mesoamerican diversity center. Genet. Resour. Crop Evol. 55: 1065–1077.

[bib33] Merotto A. Goulart I. Nunes A. Kalsing A. Markus C., 2016 Evolutionary and social consequences of introgression of non‐transgenic herbicide resistance from rice to weedy rice in Brazil. Evol. Appl. 9: 837–846. 27468302 10.1111/eva.12387 PMC4947146

[bib34] Patel R. K. Jain M., 2012 NGS QC toolkit: a toolkit for quality control of next generation sequencing data. PLoS One 7: e30619. 22312429 10.1371/journal.pone.0030619 PMC3270013

[bib35] Pusadee T. Schaal B. A. Rerkasem B. Jamjod S., 2013 Population structure of the primary gene pool of *Oryza sativa* in Thailand. Genet. Resour. Crop Evol. 60: 335–353.

[bib36] Reagon M. Thurber C. S. Gross B. L. Olsen K. M. Jia Y., 2010 Genomic patterns of nucleotide diversity in divergent populations of US weedy rice. BMC Evol. Biol. 10: 180. 20550656 10.1186/1471-2148-10-180 PMC2898691

[bib37] Rozen S. Skaletsky H., 1999 Primer3 on the WWW for general users and for biologist programmers. Methods Mol. Biol. 132: 365–386. 10.1385/1-59259-192-2:365 10547847

[bib38] Snow A. Culley T. Campbell L. Sweeney P. M. Hegde S. G., 2010 Long‐term persistence of crop alleles in weedy populations of wild radish (*Raphanus raphanistrum*). New Phytol. 186: 537–548. 20122132 10.1111/j.1469-8137.2009.03172.x

[bib39] Song B. K. Chuah T. S. Tam S. M. Olsen K. M., 2014 Malaysian weedy rice shows its true stripes: wild *Oryza* and elite rice cultivars shape agricultural weed evolution in Southeast Asia. Mol. Ecol. 23: 5003–5017. 25231087 10.1111/mec.12922

[bib40] Subudhi P. K. Parco A. Singh P. K. DeLeon T. Karan R., 2012 Genetic architecture of seed dormancy in U.S. weedy rice in different genetic backgrounds. Crop Sci. 52: 2564–2575.

[bib41] Sudinato E. Neik T. X. Tam S. M. Chuah T. Idris A. A., 2016 Morphology of Malaysian weedy rice (*Oryza sativa*): diversity, origin and implications for weed management. Weed Sci. 64: 501–512.

[bib42] Sweeney M. T. Thomson M. J. Pfeil B. E. McCouch S., 2006 Caught red-handed: *Rc* encodes a basic helix-loop-helix protein conditioning red pericarp in rice. Plant Cell 18: 283–294. 16399804 10.1105/tpc.105.038430 PMC1356539

[bib43] Sweeney M. T. Thomson M. J. Cho Y. G. Park Y. J. Williamson S. H., 2007 Global dissemination of a single mutation conferring white pericarp in rice. PLoS Genet. 3: e133. 17696613 10.1371/journal.pgen.0030133 PMC1941752

[bib52] Tajima F 1989 Statistical method for testing the neutral mutation hypothesis by DNA polymorphism. Genetics. 123: 585–595. 2513255 10.1093/genetics/123.3.585 PMC1203831

[bib44] Vigueira C. Li W. Olsen K. M., 2013 a The role of *Bh4* in parallel evolution of hull colour in domesticated and weedy rice. J. Evol. Biol. 26: 1738–1749. 23859433 10.1111/jeb.12171

[bib45] Vigueira C. C. Olsen K. M. Caicedo A. L., 2013 b The red queen in the corn: agricultural weeds as models of rapid adaptive evolution. Heredity 110: 303–311. 23188175 10.1038/hdy.2012.104 PMC3607111

[bib46] Wahab A. Suhaimi O., 1991 Padi angin characteristics, adverse effects and methods of its eradication. Teknologi Padi. 7: 21–31.

[bib47] Wang H. Xu X. Vieira F. G. Xiao Y. Li Z., 2016 The power of inbreeding: NGS based GWAS of rice reveals convergent evolution during rice domestication. Mol. Plant 9: 975–985. 27179918 10.1016/j.molp.2016.04.018

[bib48] Warwick S. I. Stewart C, 2005 Crops come from wild plants: how domestication, transgenes, and linkage together shape ferality, pp. 9–30 in *Crop Ferality and Volunteerism*, edited by Gressel J CRC Press, Boca Raton, FL.

[bib49] Xia H. B. Wang W. Xia H. Zhao W. Lu B. R., 2011 Conspecific crop-weed introgression influences evolution of weedy rice (*Oryza sativa* f. *spontanea*) across a geographical range. PLoS One 6: e16189. 21249201 10.1371/journal.pone.0016189 PMC3020953

