# Supplementary material for: Little White Lies: Pericarp Color Provides Insights into the Origins and Evolution of Southeast Asian Weedy Rice
Source: G3 (Bethesda). 2016 Oct 10;6(12):4105–14. doi: 10.1534/g3.116.035881 (PMC5144979; doi:10.1534/g3.116.035881)
Supplement: Supplemental Material [file supp_6_12_4105__index.html]

Little White Lies: Pericarp Color Provides Insights into the Origins and Evolution of Southeast Asian Weedy Rice — Supplemental Material 

# Little White Lies: Pericarp Color Provides Insights into the Origins and Evolution of Southeast Asian Weedy Rice

## Supplemental Material for Olsen *et al.*, 2016

**Files in this Data Supplement:**

- Figure S1 - Phylogenetic analysis using TCS (Phylogenetic network estimation based on statistical parsimony). Colors indicate the identity of accessions as indicated in the legend. (.pdf, 132 KB)
- Table S1 - Genotype and phenotype data for *Oryza* samples used to generate new *Rc* sequence data. (.xlsx, 29 KB)
- Table S2 - Genotype and phenotype data for *Oryza* samples previously sequenced at the *Rc* locus and included in analyses. (.xlsx, 21 KB)
- Table S3 - Comparison of SNPs differentiating the three Malaysian weedy rice groups identified in *Rc* phylogenetic analyses. (.xlsx, 37 KB)
- Table S4 - Comparison of variants within the *Rc* coding and amino acid sequences among three rice groups identified in *Rc* phylogenetic analyses. (.xlsx, 1225 KB)
- Table S5 - Nucleotide diversity for *Oryza* samples at the *Rc* locus. Values of *π, θW*, Fu and Li's F, Fu and Li's D and Tajima's D for total sites and silent sites at *Rc* locus is included. (.xlsx, 17 KB)
