# Supplementary figures and images for: Little White Lies: Pericarp Color Provides Insights into the Origins and Evolution of Southeast Asian Weedy Rice
Source: G3 (Bethesda). 2016 Oct 10;6(12):4105–14. doi: 10.1534/g3.116.035881 (PMC5144979; doi:10.1534/g3.116.035881)

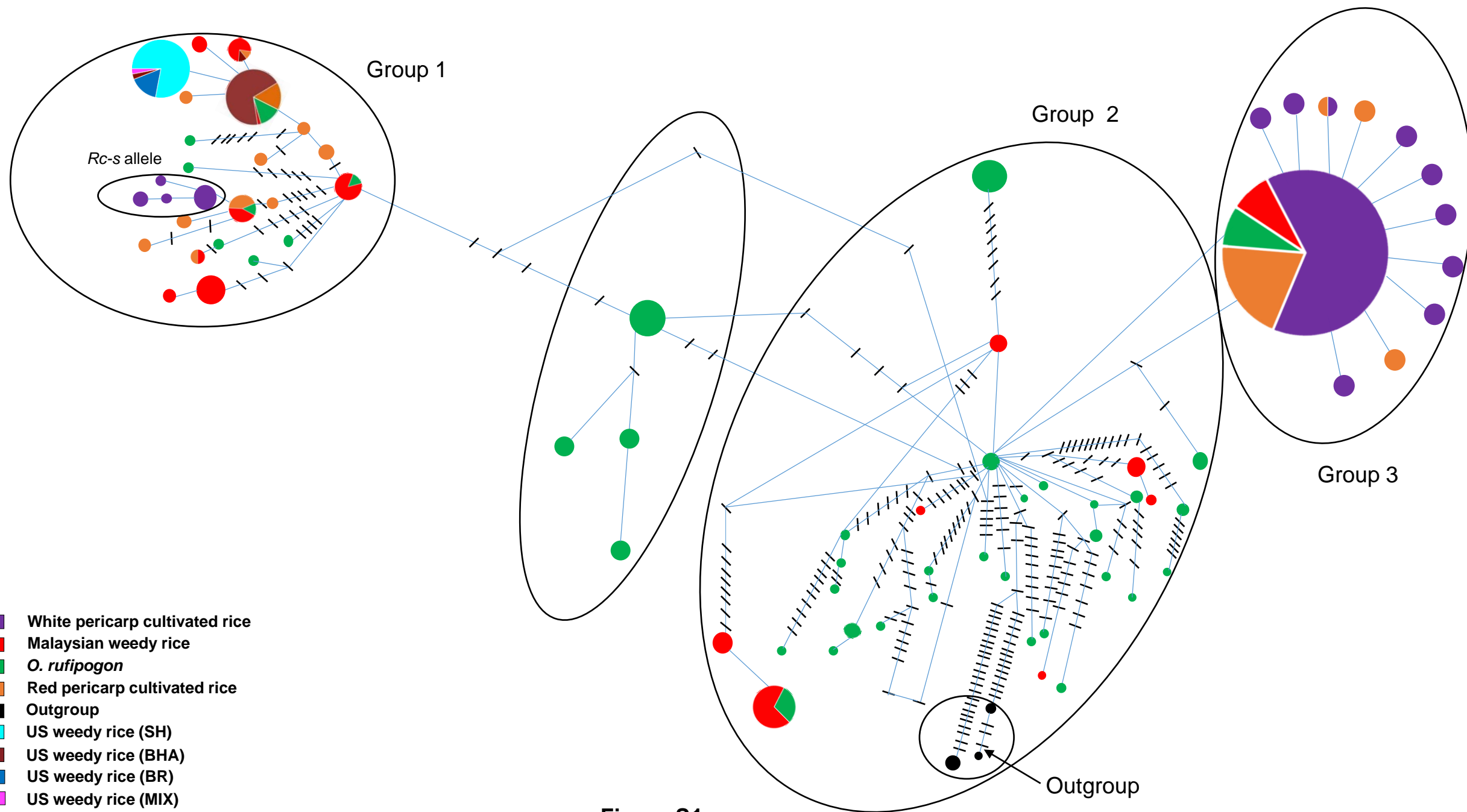

Figure S1

Supplement: Supplemental Material [file supp_g3.116.035881_FigureS1.pdf]
